# Novel Adsorbents and Adsorption Methods for Pollutant Removal

**DOI:** 10.3390/toxics11120954

**Published:** 2023-11-23

**Authors:** Yuezhou Wei, Yan Wu, Xinpeng Wang

**Affiliations:** 1 School of Nuclear Science and Technology, University of South China, Hengyang 421009, China; 2 School of Nuclear Science and Engineering, Shanghai Jiao Tong University, Shanghai 200240, China; 3 Guangxi Key Laboratory of Processing for Non-Ferrous Metals and Featured Materials, School of Resources, Environment and Materials, Guangxi University, Nanning 530004, China; wangxinpeng@gxu.edu.cn

Over the past few decades, with the rapid growth of the global population and economy, the increasing levels of various pollutants such as heavy metals, radionuclides, and organic/inorganic/biological toxins from various industries and human activities, which diffuse into aspects of the environment such as the atmosphere, soil, and natural water, have posed a serious threat to human health and the environment. Therefore, these pollutants must urgently be removed in a highly efficient and concise manner. Adsorption, as the main technology for removing pollutants from wastewater, contaminated liquids, and soil pollutants, has attracted extensive attention from researchers. In recent years, many new adsorbent materials, such as highly porous adsorbents, multifunctional hybrid materials, biomass adsorbents, nanostructured materials, and COF/MOF materials, have been developed and applied to the elimination of toxic pollutants.

This Special Issue highlights the recent progress with regard to research on novel adsorbents and adsorption methods for pollutant removal. The authors investigated the synthesis and characterization of novel adsorbent materials targeting specific pollutants in terms of their physical state, content, radiative load, and biogenicity. The evaluation of novel adsorbents was achieved through experiments on the mechanism of adsorption and thermodynamic and kinetic methods, as well as on the separation, concentration, and removal of pollutants.

A study on the separation of actinides from HLLW was investigated by Tianjiao Jiang et al. [1] This paper used an advanced process to successfully separate Am(III) from Ln(III) by setting up two separation columns using CMPO and R-BTP as the core extractants. Mohammed F. Hamza et al. [2] studied novel adsorbents of biopolymers for the mitigation of environmental pollution via heavy metals and radionuclide ions. A biopolymer was prepared as a renewable energy source via the functionalization of chitosan with sulfonic groups. A series of experiments on elemental U were carried out to study its adsorption and desorption properties. Min Huang et al. [3] reported the effect of uranium–thorium leaching from a refractory tantalum–niobium slag via micro-mineralogical analysis and roasting experiments. It is of great practical significance to reduce the radioactive hazard of waste tantalum–niobium slag and to strengthen the sustainable utilization of resources via suitable process improvement techniques.

Two studies [4,5] synthesized an anion-exchange resin and demonstrated the possibility of applying the resin to the removal of ^99^Tc elements from real radioactive waste liquids. Porous silica gel is a matrix used to support the adsorbent morphology and reduce the loss of high-value adsorbents. Yueying Wen et al. [6] prepared a KAlFe(CN)_6_/SiO_2_ adsorbent for the enrichment of Pd from HLLW. The adsorbent maintained the structural integrity below 100 kGy, and the *K*_d_ value of Pd was larger than 1625 cm^3^ g^−1^ even in 7 M HNO_3_. Xujie Chen et al. [7] presented a SiO_2_ nanofiber membrane by using the electrostatic spinning and calcination process for the removal of vaporized ^210^Po.

From the perspective of biopurification, three articles [8,9,10] discussed the purification of dioxins in soil via bioremediation technology, the purification of metal chromium in wastewater via biopolymers, and the degradation of biological pollutants via photocatalytic ternary nanocomposites. The advection and dispersion of tritiated water and pollutants such as iodine species (iodide: I^−^ and iodate: IO_3_^−^) and plutonium in deep geological repositories were studied in two respective articles [11,12]. In order to study the purification and detection technology of heavy metals in water, Dengpeng Wang, Chunlin He, and Yuling Zhao et al. [13,14,15] discussed CdSe/CdS core–shell quantum dot tracing Cd^2+^ in aqueous solutions, carbon adsorbent microwaves with waste amine–oxime removing Pb(II), and advanced Fe_3_O_4_@SFBC adsorbent removing As(III/V), respectively. Based on waste ore, two articles [16] studied the rational recovery process of radionuclides in tantalum–niobium slag and the adsorption effect of discarded coal gangue on the pollutant tetracycline hydrochloride, which reflected the important idea of turning waste into treasure.

The articles in this Special Issue demonstrate the removal of pollutants via novel adsorbents under severe conditions of different types, states, concentrations, temperatures, irradiation doses, etc., from a variety of application scenarios, such as HLLW, industrial wastewater, and the diffusion of environmental pollutants. Meanwhile, the synthesis and characterization of a variety of novel adsorbents as well as the computational methods of adsorption thermodynamics, kinetics, and product prediction based on the existing equations are also introduced.

The research on the removal methods of pollutants using new adsorbents focuses on selecting suitable methods for the removal of pollutants in complex environments, designing the structures of new adsorbents from their functions, and formulating adsorption programs that are practical and reasonable. These new adsorbents and adsorption methods will play an important role in pollutant removal and environmental remediation.
